# The impact of nutritional supplement intake on diet behavior and obesity outcomes

**DOI:** 10.1371/journal.pone.0185258

**Published:** 2017-10-09

**Authors:** Sven Anders, Christiane Schroeter

**Affiliations:** 1 Department of Resource Economics and Environmental Sociology, University of Alberta, Edmonton, Canada; 2 Agribusiness Department, California Polytechnic State University, San Luis Obispo, California, United States of America; Institut de recherche pour le developpement, FRANCE

## Abstract

After decades-old efforts to nudge consumers towards healthier lifestyles through dietary guidelines, diet-related diseases are on the rise. In addition, a growing share of U.S. consumers proactively chooses nutritional supplements as an alternative preventative way of maintaining good health, a $25.5 billion industry in the United States. This paper investigates possible linkages between the economics of consumer supplement choices and the relationship to important dietary and health outcomes. We use National Health and Nutrition Examination Survey (NHANES) data to estimate the impact of nutritional supplements intake on respondent’s body weight outcomes, controlling for diet quality.: The focus of this article is to determine whether nutritional supplements takers differ from non-takers with regard to their health outcomes when controlling for differences in diet quality, based on individual Healthy Eating Index (HEI-2010) score. The analysis applies treatment effects estimators that account for the selection bias and endogeneity of self-reported behavior and diet-health outcomes. The analysis demonstrates a negative association between supplement intake and BMI but no significant effect on an individual’s diet quality. Our findings suggest that individuals proactively invest into their health by taking nutritional supplements instead of improving diet quality through more nutritious food choices. Our results provide important contributions to the literature on a key food policy issue. Knowledge of the determinants of supplement demand in the context of strong diet-health trends should also be helpful to stakeholders in the U.S. produce sector in their competition over consumer market share.

## Introduction

Despite the proven health benefits of a diet rich in fruits and vegetables [1–6], the average U.S. adult only consumes 64% of the vegetable servings and half of the fruit servings recommended by the 2010 Dietary Guidelines for Americans (DGAs) (U.S. Department of Agriculture and U.S. Department of Health and Human Services [7]. At the same time, the consumption of solid fats, alcohol, and added sugars (SoFAAS) is 2- to 3-fold of their recommended limits [8]. The nutrient deficit from a reduced consumption of fruit and vegetables stands against the widespread intake of nutritional supplements with 62% of U.S. adults reporting to consume supplements at least occasionally and 46% are reported to take supplements regularly [9]. We solely focus on individual supplementation with nutrients vs. food fortification. Sales of dietary supplements are valued at $25.6 billion in the U.S. and form an area of strong growth and competition for the U.S. produce industry. Population ageing, retiring baby boomers and rising awareness of diet-health related disease (e.g. obesity, diabetes) are driving forces behind the expansion of preventative consumption of dietary supplements as a proactive way of maintaining good health [10]. Each year, preventative health care could save up to $43 billion, which encompasses direct medical costs and lost productivity resulting from secondary chronic health problems [11].

However, while the interplay of appropriate food choices, nutrient intakes and physical exercise in consumer health behavior and outcomes [12–14] is well documented, little is known about the role and impact of nutritional supplements as an input into consumer diet quality and health status. The 2010 DGAs state that nutrients should come primarily from food, and recommends that specific supplementation might be needed for at-risk populations, such as postpartum women, as well as older Americans [7]. However, evidence suggests that the intake of nutritional supplements may be unnecessary and potentially even be detrimental to human health [15–16]. As such, the 2015 Dietary Guidelines Advisory Committee (DGAC) emphasizes that healthy dietary patterns are to be achieved through recommended food and beverage choices rather than with nutritional supplements except as needed for at-risk populations [17]. These inconsistencies highlight the need for research that expands the understanding of the role of nutritional supplements in U.S. consumer’s diet-health behavior and whether supplements are currently replacing or supplementing a healthy diet. Consumers may not have access to complete information about the costs and benefits of supplements and their potential effects on diet quality and personal health [18].

This article provides an important research contribution by estimating the relationship between health behavior and its potential linkage to dietary quality outcome measures, utilizing the case of nutritional supplements intake. Our objectives are to identify and quantify (1) determinants of nutritional supplements intake decisions (2) whether and to what extent supplement takers and non-takers differ with regard to diet-health outcomes (e.g. Body Mass Index (BMI)) when differences in diet quality (as measured by the Healthy Eating Index (HEI) are controlled for, and (3) whether and to what extent supplement takers and non-takers differ in diet quality (HEI) outcomes when differences in BMI are controlled for.

Previous studies acknowledge the interdependence of health behavior, dietary choices and health outcomes in terms of their short- and long-term public health impacts [19–23]. However, apart from a few exemptions [24–25] the literature on diet-health and behavior typically neglects to incorporate explicit measures of diet or health or does not account for the possible endogeneity of the determinants of behavior. A common limitation is that key determinants of diet-health behavior such as socio-economic factors and unobserved heterogeneity may simultaneously influence individuals’ behavior and the stock of diet-health. Consequently, empirical estimates of behavior and the effects of exogenous factors will be biased, potentially leading to misguided policy conclusions. Such bias can be avoided by treating direct measures of diet-health behavior as endogenous determinants of health outcomes and by adopting appropriate modelling procedures to avoid this endogeneity bias and related measurement error.

The analysis in this article builds on [22], to our knowledge the only study that incorporates health indicators and other lifestyle variables into the study of nutritional supplements intake and food quality. We expand on this topic using a more recent dataset from the 2007–2008 NHANES and updated 2010 Healthy Eating Index (HEI-2010) scores. To overcome the issues of endogeneity and measurement error resulting from the possible self-selection bias in the NHANES data, our approach employs propensity score matching (PSM) estimators to determine the possible link between nutritional supplements intake, food quality and obesity outcomes. Nutritional supplements intake does not directly affect the BMI, yet, it might impact food quality choices, which may in turn influence the BMI.

PSM has emerged as a popular approach in the estimation of causal treatment effects in economic analyses. Given the reliance of the diet-health behavioral literature on cross-sectional observational data, such as NHANES, the analysis of treatment effects is often complicated by non-linear relationships and limited dependent outcome variables that are possibly endogenous. Compared against established analytical techniques including fixed effects models [26], Heckman-type switching regression modeling [27], and difference-in-difference estimators [28], PSM methods have been shown to be superior in eliminating the biases resulting from endogenous determinants and self-selection in ensuring the comparability of different groups in the process of outcome evaluations [29–30].

From a policy standpoint, it is important to understand what factors drive consumers’ compliance with nutritional recommendations [23] and what factors might impact an individual’s decision to consume nutritional supplements as likely substitutes in meeting specific diet quality and health outcomes. Results from our study will help to develop a better understanding of the factors that affect nutritional supplements intake as an input into the development of more efficient and effective promotional strategies for healthy food choices and targeted consumer health education.

## Methodology

Economists have long been interested in the study of the interdependencies between dietary choices, nutrition and health outcomes in terms of their short- and long-term impacts on diet patterns and public health outcomes [31]. Becker’s model of investment in human capital [32] and Grossman’s seminal work on health capital [33–34] formalize the process by which individuals are endowed with a certain stock of health that deteriorates over a person’s lifetime [35–37]. The deterioration speed of a person’s health status depends, among other things, on investments in health through certain health behaviors.

A diet that follows the recommendations of the 2010 DGAs could be considered as an investment into an individual’s health stock and consuming the recommended amount of fresh fruits and vegetables as an investment in health. If an individual substitutes or complements the fruit and vegetable intake with nutritional supplements, the latter would constitute a similar investment in health capital, given that supplements may contribute to the overall utility derived from good health. Consumption choices such as smoking, alcohol intake, lack of exercise, and poor dietary patterns could accelerate the depletion rate of a person’s health stock. The depletion of the health stock beyond a certain threshold is associated with a higher probability of early death.

There are many intertemporal utility functions that could serve as a theoretical model for our analysis, such as the one developed by [33–34]. The empirical analyzes of individual’s diet behavior in the context of specific health outcomes is typically complicated by potential endogeneity between key variables of interest and a measurement error resulting from self-selection bias, which is an issue often encountered in consumer survey studies. Due to potential misspecification errors, the use of ordinary least squares estimators (OLS) may lead to biased results [38]. Instrumental variable estimators (IV) form a common econometric solution to minimize endogeneity. However, their application is often constrained by the availability of suitable instruments [39].

In this study, the nature of the NHANES data and the specific research questions make it even more difficult to find suitable instruments. For these reasons, common IV approaches are deemed less suitable. PSM, originally developed by Rosenbaum and Rubin (1983), has enjoyed increasing popularity in empirical studies of situations where the effect and outcome of a specific treatment is of interest [25,40–41]. In the economics literature, PSM has been employed to determine the effects of labor market and training courses on individual’s wage earnings [42–44]. In health economics and food consumption studies, PSM methods have been utilized to analyze how consumers that were exposed to a particular treatment (e.g. food label usage) differed from those who reportedly did not receive the same treatment [25,45–46]. In our study, PSM will account for the potential selection bias of the self-reported nutritional supplements intake and possible endogeneity of the supplement intake in the treatment outcome variable.

### Theoretical model

The rationale behind the PSM approach is to assess the effect of receiving treatment from a pool of treated and non-treated individuals. In this article, consumers who took nutritional supplements during the past 30 days will be referred to as the treatment group (supplement takers) and those who did not consume any supplements will form the control group (non-takers). We define nutritional supplement intake following [47] as any supplements and/or use of multivitamins/multi-minerals (defined as a product containing 10 vitamins and/or minerals), and/or use of individual vitamins, minerals, and non-vitamin, non-mineral supplements by an individual during the reporting period (also see NHANES data documentation for ‘Dietary Supplement Use 30-Day’; [48]). The propensity score will describe the conditional probability of taking nutritional supplements, given equality in pre-treatment characteristics between both groups. This relationship can formally be expressed as:

p(X)≡Pr(D=(1|X)=E((D|X), (1)

where *D* represents the intake of nutritional supplements (taker = 1, non-taker = 0), and *X* is a vector of pre-treatment characteristics (e.g. gender). If the health outcomes are *Y*_*0i*_ and *Y*_*1i*_ for non-takers and supplement takers, respectively, then the treatment effect for an individual ‘i’ can be written as:

$$t_{i}=Y_{li}-Y_{01i}.$$ (2)

The propensity score can be estimated with any standard probability model. The population average treatment effect (ATE) and the average effect of treatment on the treated (ATT) are the two commonly cited parameters of interest in literature and are given by:

τATE=E(τ)=E[Y(1)−Y(0)] (3)

τATT=E((τ|D)=1)=E[Y((0)|D)=0]. (4)

*Y(1)* and *Y(0)* are the two possible outcomes with and without supplement intake. The parameter of interest is the average treatment effect on the treated (ATT), because it gives the difference between expected outcome values of supplement takers and non-takers. Estimating the average treatment effect on the treated is only possible under certain assumptions, because the counterfactual is not observed. Several assumptions need to hold in order to obtain reliable treatment effects using PSM.

The first assumption is balancing the pre-treatment variables on a given propensity score [25,41,49]. Thus, for a given propensity score, nutritional supplements takers and non-takers are assumed to have closely matching distributions of observable characteristics *X*, irrespective of their treatment status. This ensures that treatment is random and takers and non-takers are observationally random.

D⊥X ∣p(X), (5)

where, *p(X)* is the propensity score. This implies that all variables that influence treatment assignment and potential outcomes simultaneously have to be observed by the researcher.

The next assumption is usually referred to as ‘unconfoundedness’ or ‘conditional independence’ assumption (CIA) [41,49–50].

Y1,Y0⊥D∣p(X). (6)

This assumption implies that potential outcomes are not dependent on treatment. In other words, variables that can affect both treatment and potential outcomes concurrently have to be observed by the researcher. Another assumption is that of ‘overlap’ [41,49] given as;

0<P(D=1∣X)<1. (7)

This assumption ensures that individuals with the same characteristics *X* (e.g. income level) are assumed to have an equal chance of being part of the treatment or control group. Once the above assumptions are satisfied, the propensity score of the ATT can then be estimated reliably. To further validate whether our selections models meet the assumption of conditional independence, in other words, whether and to what extend unobserved variables in the treatment selection model may bias the estimates of subsequent treatment effects we perform Rosenbaum bounds tests [51]. The bounds test statistics allows us to assess the strength unmeasured or unobserved selection variables must have in order that the estimated treatment effects from propensity score matching would have resulted from a purely non-random assignment [52].

### Empirical models

The analysis in this article employs data from the 2007–2008 National Health and Nutrition Examination Survey (NHANES) (Centers for Disease Control and Prevention [48]. The NHANES is the primary national survey used to assess the health and nutritional status of the U.S. population. Participants in the NHANES are randomly selected civilian residents of the United States. The survey is divided into the physical examination, questionnaire and personal interview components. The interview is used to gather information on demographic, socioeconomic, nutritional, and health related issues. The physical examination component is generally used to conduct laboratory investigations [48].

Data from various NHANES survey cycles has been used in a number of similar studies focused on individual’s health behavior, food consumption choices, and a multitude of other economic and non-economic research questions [16,19–22,53–54]. For the purposes of the analysis in this article, only adult NHANES participants of at least 20 years were selected, as this sample typically makes their own food, diet or health behavioral (e.g. nutritional supplements intake) decisions.

From the large pool of available NHANES variables, we selected a number of relevant observables directly associated with the treatment of interest: nutritional supplements intake (treatment), diet quality and health indicators (outcomes), demographics, and different relevant lifestyle determinants (S1 Table supporting information). We expect this range of variables to minimize unobserved heterogeneity among NHANES respondents that may influence individuals’ diet-health behaviors and thus BMI and diet quality outcomes of interest to us. The empirical PSM selection models to be estimated are specified as:

$$Supplement=f\left(\begin{array}{c} HEI,\;Diabetes,\;Blood\;pressure,\;Male,\;Age,\;White,\\ Hispanic,Other\;race,Citizen,\;Household\;size,\\ Married,\;Divorced,High\;school,\;Graduate,\;HHInc2,\\ HHInc3,\;HHInc4,HHInc5,\;Food\;stamp,\\ Smoker,\;Alcohol,\;Very\;active \end{array}\right),$$ (8)

and

$$Supplement=f\left(\begin{array}{c} BMI\;,\;Diabetes,\;Blood\;pressure,\;Male,\;Age,\;White,\\ Hispanic,Other\;race,Citizen,\;Household\;size,\\ Married,\;Divorced,High\;school,\;Graduate,\;HHInc2,\\ HHInc3,\;HHInc4,HHInc5,\;Food\;stamp,\\ Smoker,\;Alcohol,\;Very\;active \end{array}\right),$$ (9)

where *Supplement* is a binary dependent variable that indicates that the individual has consumed nutritional supplements in the past 30 days.

The Healthy Eating Index (*HEI*) is a tool used to measure the diets of Americans against the DGAs. The HEI is composed of twelve sub-components such as HEI Total Fruits, HEI Total Vegetables, HEI Greens & Beans, which carry individual scores that add up to hundred to give the Total HEI. A higher HEI score indicates a diet of higher quality. Using the code written by [54], we computed the Healthy Eating Index-2010 (HEI-2010) for all NHANES participants in our sample. A negative relationship between nutritional supplements intake and BMI outcomes has been documented in previous research and is of particular to food policy [21].

With regard to the variables Diabetes and Blood Pressure, previous literature shows a controversial relationship between these health conditions and nutritional supplements intake. Some reports show no association while others have documented a negative impact [19,55–57].

Based on previous research, we expect supplement intake to be positively associated with education, income, female, age and white [21,58–61]. Lifestyle factors such as smoking, alcohol intake are expected to have a negative relationship with nutritional supplements intake [21,59,62]. In contrast, those following an active lifestyle (e.g. very active) are assumed to be more likely to consider supplements as part of their health behavioural choices. We anticipate that food stamps recipients might form an at-risk population and may need supplements to boost their diet quality.

An ad-hoc approach to the matching of individuals in order to achieve an optimal balancing of pre-treatment characteristics is unfeasible [41]. Instead, our selection of variables in building the propensity score model in Eq (8) is guided by economic theory and a sound assessment of previous relevant research. Accordingly, our first step of analysis involves the estimation of Eq (8) to achieve the critical identification assumption of unconfoundedness (CIA), a necessary step for the unbiased estimation of treatment effects. The resulting balancing of covariate variables between treatment and control group members is then conveniently expressed in an individual’s propensity score as a single-index variable input into the second-stage matching procedure. Matching algorithms commonly applied in PSM studies are: Nearest Neighbor, Caliper (Radius), Stratification and Kernel matching algorithms. The estimation of propensity scores and matching algorithms is performed using the psmatch2 package in Stata [63].

## Results

A key feature of the propensity score matching approach is its ability to reduce the self-selection bias and resulting measurement error in treatment effects. In order to validate the quality of matching between nutritional supplements takers and the counterfactual group of non-takers, we perform Rosenbaum’s (1991) [64] standard bias test (see S2 Table supporting information). By comparing the difference of the sample means in the treated and matched control sub-samples for each covariate (see S3 Table supporting information), expressed as a percentage of the square root of the average of the sample variances in both groups, the test allows us to quantify the reduction in selection bias and the quality of the chosen covariate in the propensity score model. Examining the t-test results of unmatched and matched covariates reveals insignificant differences in the matched samples after the propensity score estimation. We also evaluate minor changes in our model specification. Our results are largely insensitive to alternative variables, with the visible exemptions of a few variables (e.g. HEI-Dairy). Overall, the results on matching quality imply that our propensity score specification is reliable and robust. Both propensity score models satisfy the balancing hypothesis (common support), allowing us to test whether nutritional supplement generate significant differences in our selected diet quality and obesity outcomes. In addition, S2 Table presents the mean value of the standard bias measure across the different matching algorithms. For the impact of supplement on NHANES participants BMI the mean standard bias before matching is roughly 12%. PSM is able to reduce this bias significantly for all matching algorithms to levels between 1.2% and 2.7%; a range generally considered reliable [41].

The focus of this article is to determine whether nutritional supplements takers differ from non-takers with regard to their health outcomes when controlling for differences in diet quality. Supplements are assumed to contribute to an individual’s utility derived from good health and are inputs to the person’s health production function. The factors associated with diet-health behavior and specifically nutritional supplements intake decisions are diet quality, health indicators, demographics, and lifestyle. In order to identify and quantify the determinants of supplement intake decisions, the PSM model in (8) was estimated to match all the respondents on a wide range of variables. Table 1 shows the factors associated with the selection into the treatment group of supplement takers.

**Table 1:** Determinants of dietary supplement intake.

Variables (Y = Supplement)	Coefficients	Standard Error
***Diet Quality***		
HEI-Total vegetables	-0.282	2.906
HEI-Greens & beans	0.122	0.955
HEI-Total fruits	0.840	1.206
HEI-Whole fruits	-0.957	0.743
HEI-Whole grain	0.689	2.088
HEI-Dairy	-0.511	0.745
HEI- Seafood & plant protein	-0.383	0.571
HEI- Fatty acid ratio	-0.617	0.851
HEI-Sodium	0.658	0.528
HEI-Refined grains	0.727	0.537
HEI-SoFAAS calories	0.117	0.480
***Health indicators***		
Diabetes	-0.026	0.094
Blood pressure	-0.189	0.243
***Demographics***		
Male	-0.590***	0.066
Age	0.032***	0.002
White	0.444***	0.084
Hispanic	0.336***	0.119
Other race	0.230**	0.104
Citizen	0.458***	0.113
Household size	-0.090***	0.023
Married	0.113	0.096
Divorced	0.018	0.112
High school	-0.032*	0.075
Graduate	0.403***	0.088
HHInc2	0.202**	0.084
HHInc3	0.324***	0.091
HHInc4	0.488***	0.120
HHInc5	0.694***	0.112
***Lifestyle***		
Food stamps	-0.218***	0.076
Smoker	-0.165***	0.063
Alcohol	0.101	0.065
Very active	0.491***	0.085
Constant	-6.458	5.966
Number of observations	5,063	
Log-likelihood	-3102.18	
Pseudo R^2^	0.114	

*** indicates significance at the 99% level.

** indicates significance at the 95% level.

* indicates significance at the 90% level.

The common support criterion was imposed to assure maximum overlap between propensity scores of control and supplement taker group (Heckman, Ichimura, and Todd, 1998).

Table 2 indicates no relationship between health indicator variables and nutritional supplements intake. Previous literature shows mixed results with regard to supplement intake and the presence of a health condition like diabetes or high blood pressure. While some of the studies report that there is a negative relationship between supplement intake and *diabetes* and *blood pressure* [56–57], others conclude that there is no association between supplement intake and these conditions [19,55]. In addition, we found no association between selection into the nutritional supplements intake group and all the components of the HEI-2010.

**Table 2:** Determinants of selection into dietary supplement intake group.

Variables (Y = Supplement)	Coefficients	Standard Error
***Health indicators***		
BMI	-.0136***	0.005
Diabetes	0.0522	0.097
Blood pressure	-0.210	0.243
***Demographics***		
Male	-0.580***	0.0661
Age	0.0348***	0.002
White	0.399****	0.084
Hispanic	0.342***	0.120
Other race	0.262**	0.105
Citizen	0.340***	0.114
Household size	-0.0749***	0.023
Married	0.115	0.097
Divorced	0.0129	0.113
High school	0.333***	0.088
Some college	0.708***	0.089
Graduate	0.823***	0.104
HHInc2	0.157*	0.085
HHInc3	0.239***	0.092
HHInc4	0.378***	0.122
HHInc5	0.571***	0.114
***Lifestyle variables***		
Food stamps	-0.161**	0.074
Smoker	0.399***	0.084
Alcohol	0.0920	0.065
Very active	0.440***	0.086
Constant	-2.080***	0.347
Number of observations	5063	
Log-likelihood	-3072.87	
Pseudo R^2^	0.122	

*** indicates significance at the 99% level.

** indicates significance at the 95% level.

* indicates significance at the 90% level.

The common support criterion was imposed to assure maximum overlap between propensity scores of control and supplement taker group (Heckman, Ichimura, and Todd, 1998).

Table 2 shows that with the exception of marital status and high school, all of the demographic factors are significant at explaining the probability of being selected into the treatment group. Demographic factors that positively affect the probability of taking nutritional supplements are *age*, *ethnicity*, a higher level of *education*, and a higher *household income*. These results conform to previous research [21,53,59–61,65]. We find that *males* are 59% less likely to take nutritional supplements compared to their female counterparts. This finding confirms the results of previous studies [21,59,62,65]. The negative relationship between *male* and supplement intake suggests that females might be more concerned about diet behavior. Our findings suggest that ethnic heritage seems to play an important role in determining selection into the treatment group. In comparison to *African American* individuals, individuals of *other races* are more likely take nutritional supplements.

The negative effect of *household size* on nutritional supplements intake suggests that members of larger households may not consume supplements, given budgetary constraints [62]. Consumers who completed a higher level of *education* may be in a more informed position to take control of their health. Participants who fall in the highest income group, often correlated with higher educational attainment have the greatest propensity (69%) to take supplements, which confirms an income and educational gradient in that nutritional supplements intake decisions reported in previous studies [47,65].

Our results for the lifestyle category show that *food stamp* recipients are 22% less likely to take nutritional supplements compared to other respondents. Food stamps may not be used for the purchase of vitamins and supplements [66]. Our result suggests that nutritional supplements are not consumed by one important target group of at-risk consumers who may be in need of complementary supplementation with nutrients.

As has been commonly found in previous related literature [22,57,62,65,67–69], *smokers* are 17% less likely to take nutritional supplements as compared to non-smokers. This negative relationship may indicate that *smokers* are less concerned about their health. Our explanation for the sign change in the smoker variable indicates that smokers tend to be less concerned about their health. However, Table 2 shows that smokers who take supplements tend to have lower BMIs. This confirms the findings of previous research that shows that smokers tend to have lower BMIs on average, compared to non-smoking population. Using NHANES data from the same time frame, Plurphanswat and Rodu (2014) [70] show that both male and female smokers are more likely to be underweight and normal weight compared to never smokers. We did not find any significant relationship between the heightened consumption of *alcohol* and taking nutritional supplements. Previous research shows that the health impact of alcohol on diet quality is ambiguous [71].

Individuals who exhibited active lifestyles are 49% more likely to take nutritional supplements. This is consistent with findings from previous literature [20, 55,57,62,65,69,72–73].

### Analyzing health outcomes of nutritional supplements consumers

In order to deepen the PSM analysis, we used different matching algorithms to build on the estimated PSM model in order to determine whether regular consumers of nutritional supplements may display improved health outcomes, as measured by their BMI. Thus, we aimed at quantifying whether and to what extent supplement takers and non-takers differ in BMI outcomes when variations in diet quality (HEI) are controlled for. We used the factors discussed in Table 1 to determine the selection into the treatment group. Table 3 shows the average ATTs applying different matching algorithms for the comparison of respondents in the nutritional supplements treatment group versus the control group.

**Table 3:** Average effect of treatment on the treated (ATT) for dietary supplement intake on BMI.

Matching Algorithm	Coefficient	Standard Error^1^
Nearest Neighbor Matching	-1.480***	0.316
Radius Matching (r = 0.1)	-1.150***	0.221
Radius Matching (r = 0.001)	-1.234***	0.238
Kernel Matching	-1.141***	0.210
Stratification Matching	-1.071***	0.237

Notes:

***p < .001.

** p < 0.05.

* p < 0.1.

^1^ Bootstrapped standard errors of ATT estimates using 100 repetitions.

The results in Table 3 show a clear distinction between nutritional supplements takers and non-takers in terms of their BMI. Our results suggest that the individual HEI components do not have a significant impact on supplement intake. Despite this lack of significance, there exists a relationship between supplement intake and total HEI and thus, BMI. The consistent outcome across all the matching algorithms is worth noting: Across the select matching algorithms, supplement takers have a lower BMI of more than 1 kg/(body height in m).^2^

The significant difference in BMI between nutritional supplements takers and non-takers is striking, because the components of the HEI-2010 did not have a significant effect on the selection into the treatment group. Our results expand the findings of previous studies that have found inconclusive results [25]. According to Kimmons et al. [74] individuals who are obese or overweight are less likely to take nutritional supplements. Balluz et al. [19] note that those who are overweight or obese may have a greater tendency to take supplements because they may be making weight loss attempts or are on a special diet that may include nutritional supplements.

### Nutritional supplements intake and diet quality

In order to quantify whether and to what extent supplement takers and non-takers differ in diet quality (HEI) outcomes when differences in BMI are controlled for, we repeated the matching procedure while controlling for differences in BMI. Table 1 shows the determinants for selection into the treatment group of nutritional supplements taker. In addition to using the variables presented in Table 1, we added the variables *BMI* and *some college* into our model.

The introduction of another education variable resulted in all of the education variables becoming significant at explaining the selection into the treatment of group of being a nutritional supplements taker. *BMI* has a significant negative relationship on selection into the treatment group. Previous research has documented the negative relationship between BMI and nutritional supplements intake [21,60,62,67,69,72–75].

We calculated ATTs to determine whether significant differences exist between supplement takers and non-takers in terms of HEI. Furthermore, we selected three sub-component scores of the HEI-2010 (*HEI Total*, *HEI Total Vegetables* and *HEI Total Fruits*) due to the known relationship between fruit and vegetables intake and obesity. Table 4 shows the results of the various matching algorithms.

**Table 4:** Average effect of treatment on the treated (ATT) for dietary supplement intake on Healthy Eating Index (HEI) and select subcomponents^1^.

HEI	Nearest NeighborMatching	Radius Matching		Kernel Matching	Stratification Matching
		(R = 0.1)	(R = 0.001)		
**Total**	0.0813* (0.048)	0.0341 (0.035)	0.0432 (0.041)	0.0514 (0.034)	0.0596* (0.035)
**Total vegetables**	0.0047 (0.003)	0.0013 (0.002)	0.0019 (0.003)	0.0023 (0.002)	0.0029 (0.003)
**Total fruits**	0.0125* (0.007)	0.0072* (0.004)	0.0090* (0.005)	0.0092** (0.004)	0.0103*** (0.003)
**SoFaas calories**					

Notes:

***p < .001.

** p < 0.05.

* p < 0.1.

^1^ Bootstrapped standard errors of ATT estimates using 100 repetitions in brackets.

For the nearest Neighbor matching method and stratification matching, we find a significant positive relationship between *HEI total* and nutritional supplements intake confirming previous results by Schroeter, Anders, and Carlson [22] and Kennedy [76]. Table 4 also shows a higher score of *HEI Total Fruit* for supplement consumers However, while both results indicate that supplement consumers have overall higher diet-quality scores the magnitudes of the effects remain insignificant. Finally, we did not find any difference for the *HEI Vegetables* between nutritional supplements takers and non-takers.

Finally, propensity-score matching estimators critically hinge on the assumption of unconfoundedness or CIA. Hence, PSM cannot provide consistent treatment estimates if the assignment to treatment is endogenous; such that unobserved variables critically affect the assignment process and related outcome estimates. In order to estimate the extent to which potential unmeasured and/or unobserved factors may bias our treatment effects, we conducted Rosenbaum bounds sensitivity analysis on both HEI and BMI models [51]. The bounds tests validate the estimated ATTs, while setting the level of hidden bias to a certain value, allowing us to directly asses the required size (or strength) of the unobserved selection variables such that the estimated ATTs would violate the CIA condition. Based on Wilcoxon signed rank tests, the results suggest that the average treatment effects in Tables 3 and 4 and underlying propensity score models in Tables 1 and 2 are robust against the hidden bias of unobserved selection variables. The estimated values of hidden bias needed to render our effects estimates a non-random range around 1.1, i.e. a value confirmed to indicate robustness in the literature [52]. The test results are available from the author upon request.

## Conclusions

Our study shows that the propensity to consume nutritional supplements is a function of diet quality, health, demographic and lifestyle factors. Our findings also suggest a possible link between diet-health behavior (supplement intake) and obesity status as measured by BMI. Thus, consumers of diet supplements do show a lower BMI compared to non-takers. Given decreasing intake levels of fruits and vegetables, it is important to determine the role nutritional supplements play in health behavior and in determining diet quality outcomes.

Indeed, we find that nutritional supplements intake may have a positive effect on diet quality of supplement consumers, which in turn may affect diet health outcome indicators, such as the BMI. However, the effect of supplement intake as an explicit health behavior does not significantly alter the levels of overall diet quality or any of its important sub-components observed in this study. This finding expands the results commonly found in consumer stated preference surveys on diet-health in that a direct linkage between preventative health behaviors and the consumption of fruits and vegetables exists [10].

However, the estimated differences in obesity and diet quality outcomes between nutritional supplement takers and no-takers should not be interpreted as the causal effect of supplementation. Instead, the binary supplement intake variable in the PSM analysis represents a proxy of individual’s (supplement taker’s) health behaviour, which results in significant differences in the chosen outcomes when compared against a control (non-taker) population.

The results of the analysis suggest that several health indicators, demographics, and lifestyle variables significantly affect the selection into the treatment group of nutritional supplement takers. Nutritional supplement intake is positively associated with a significantly lower BMI of above 1kg/ (body height in meters)^2^, when all other observable characteristics between supplement takers and non-takers are controlled for. We also found that supplement takers are likely to be white, highly educated, of higher household income, non-smokers and of overall higher health status.

The findings reveal that food stamp recipients and lower income households do not take nutritional supplements, even though these two groups may be especially at-risk groups of failing to meet recommended intake levels for major fresh food categories (e.g. fresh fruits and vegetables). On the other hand, individuals of normal weight (individuals with a lower BMI) and individuals who consume more fruits were found to proactively hedge against health risks by consuming nutritional supplements a preventative measure for maintaining good health. One way to encourage consumption of nutritional supplements among at-risk groups would be to establish a health policy on consumption, especially with regards to fruits and vegetables and nutritional supplements, in order to target specific at-risk populations.

Given the increasing importance of individuals’ dietary choices to consumer diet-health and public policy in the United States, accurate estimates of existing behaviors and their impacts on relevant health outcomes have become essential tools for the purpose of policy guidance. Moreover, greater general awareness of diet-health issues and trends toward proactive health behaviors have increased consumers’ demand for more products with identifiable health benefits (e.g. superfoods, functional foods). This trend towards “nutritionism” [77] demands significant adjustments on the side of stakeholders along many agri-food value chains. Given the recent volatility in the in the U.S. produce sector, growth opportunities related to diet-health trends should be of even greater important to the fresh fruit and vegetable industry. In this context knowledge of the determinants of nutritional supplement demand seems particularly essential.

Finally, a key component in the quest for improving food policies is the improvement in analytical methods aimed at eliminating the self-selection bias and resulting mismeasurement commonly associated with working with cross-sectional observational data, such as NHANES. The econometric analysis carried out in this article contributes to the discussion regarding whether consuming supplements leads to positive diet-health outcomes. Appropriate methods such as treatment effects estimators (e.g. PSM) can provide more reliable insights into an individual’s diet and health behavior, which will provide the prerequisite for effective and efficient public policies.

## Supporting information

S1 Table Descriptive statistics of variables. (DOCX)

S2 Table Standard bias for different matching algorithms. (DOCX)

S3 Table Reduction in self-selection bias and covariate balancing. (DOCX)

S1 Dataset (XLSX)
