# Supplementary material for: The impact of nutritional supplement intake on diet behavior and obesity outcomes
Source: PLoS One. 2017 Oct 9;12(10):e0185258. doi: 10.1371/journal.pone.0185258 (PMC5633155; doi:10.1371/journal.pone.0185258)
Supplement: S1 Table — (DOCX) [file pone.0185258.s001.docx]

**Table B:** Descriptive Statistics of Variables

| Variable | Description | Suppl=1 | Suppl=0 |
| --- | --- | --- | --- |
|  |  | Mean (St.dev.) | Mean (St.dev.) |
| *Dietary supplement intake* | | | |
| Supplement | = 1 if respondent has taken any dietary supplements in the past 30days | 1  (0) | 0  (0) |
| *Diet Quality* | | | |
| HEI Total | Total Healthy Eating Index 2010 (HEI-2010) | 54.63  (1.08) | 54.58  (1.09) |
| HEI-Total vegetables | HEI-2010 for total vegetable | 3.15  (0.08) | 3.15  (0.08) |
| HEI green beans | HEI-2010 for greens and beans | 2.19  (0.19) | 2.19  (0.19) |
| HEI total fruit | HEI-2010 for total fruit | 3.07  (0.14) | 3.07  (0.15) |
| HEI whole fruit | HEI-2010 for total whole fruit | 4.11  (0.21) | 4.10  (0.22) |
| HEI whole grains | HEI-2010 for total whole grains | 1.95  (0.09) | 1.95  (0.09) |
| HEI dairy | HEI-2010 for total dairy | 5.58  (0.15) | 5.58  (0.15) |
| HEI SFPP | HEI-2010 for total seafood and plant proteins | 3.00  (0.13) | 2.99  (0.12) |
| HEI far | HEI-2010 for total fatty acid ratio | 3.97  (0.11) | 3.96  (0.11) |
| HEI na | HEI-2010 for total sodium | 4.35  (0.13) | 4.35  (0.13) |
| HEI rg | HEI-2010 for total refined grains | 6.78  (0.13) | 6.77  (0.13) |
| HEI sc | HEI-2010 for total empty calories | 11.49  (0.35) | 11.48  (0.36) |
| *Health indicators* | | | |
| Body Mass Index | =Weight (kg)/ (Height (m))^2^ | 28.64  (6.37) | 29.25  (6.90) |
| Diabetes | =1 if respondent has been told by doctor or health professional to have diabetes | 0.13  (0.34) | 0.12  (0.32) |
| Blood pressure | =1 if respondent has been told by doctor or health professional to have high blood pressure | 0.96  (0.19) | 0.95  (0.22) |
| *Demographics* | | | |
| Male | =1 if respondent is male | 0.44  (0.50) | 0.54  (0.50) |
| Age | Age of respondent in years | 55.26  (17.18) | 46.16  (17.25) |
| White | =1 if respondent is non-Hispanic white | 0.55  (0.50) | 0.39  (0.49) |
| Black | 1 if respondent is non-Hispanic Black | 0.17  (0.38) | 0.23  (0.42) |
| Hispanic | =1 if respondent is Hispanic | 0.10  (0.30) | 0.12  (0.33) |
| Other race | =1 if respondent is none of the races above | 0.17  (0.38) | 0.25  (0.43) |
| Citizen | =1 if respondent was born in the USA | 0.92  (0.27) | 0.82  (0.38) |
| Household size | Total number of individuals in household | 2.81  (1.53) | 3.42  (1.72) |
| Married | 1 if respondent is married/common law | 0.62  (0.49) | 0.58  (0.49) |
| Divorced | =1 if respondent is divorced or separated | 0.26  (0.44) | 0.21  (0.41) |
| Single | =1 if respondent is single/never married | 0.12  (0.33) | 0.21  (0.41) |
| High school | =1 if respondent went to high school | 0.23  (0.42) | 0.26  (0.44) |
| Some college | =1 if respondent went to some college | 0.29  (0.45) | 0.23  (0.42) |
| Graduate | =1 if respondent graduated from college and above | 0.25  (0.44) | 0.13  (0.33) |
| HHInc1 | =1 if annual household income 0.35(0.48) is between $0-$24,999 | 0.30  (0.46) | 0.39  (0.49) |
| HHInc2 | =1 if annual household income is between $25000-$49,999 | 0.21  (0.41) | 0.22  (0.42) |
| HHInc3 | =1 if annual household income is between $50,000 - $ 74,999 | 0.19  (0.39) | 0.18  (0.39) |
| HHInc4 | 1 if annual household income between $75,000 - $ 99,999 | 0.10  (0.30) | 0.08  (0.26) |
| HHInc5 | 1 if annual household income is $100,000 and over | 0.17  (0.37) | 0.09  (0.28) |
| *Lifestyle* | | | |
| Food stamp | 1 if respondent has ever received food-stamps | 0.17  (0.40) | 0.31  (0.54) |
| Smoker | =1 if respondent has smoked at least 100 cigarettes in entire life and is currently smoking | 0.45  (0.51) | 0.51  (0.60) |
| Alcohol | Alcohol =1 if respondent has consumed at least 12 alcoholic beverages in last year | 0.70  (0.55) | 0.72  (0.48) |
| Very active | =1 if respondent’s self-rated daily activity is very vigorous | 0.17  (0.37) | 0.22  (0.41) |

Note: Descriptive statistics based on unmatched HNHANES sample data.
