# Supplementary material for: The impact of nutritional supplement intake on diet behavior and obesity outcomes
Source: PLoS One. 2017 Oct 9;12(10):e0185258. doi: 10.1371/journal.pone.0185258 (PMC5633155; doi:10.1371/journal.pone.0185258)
Supplement: S2 Table — (DOCX) [file pone.0185258.s002.docx]

**Table:** Standard Bias for different matching algorithms

| **Before matching** | |
| --- | --- |
| Mean absolute bias | 11.9 |
| Pseudo R^2^ | 0.114 |
| LR χ^2^ (p-value) | 797.31 (0.00) |
| ***Nearest Neighbor*** | |
| Mean absolute bias | 1.6 |
| Pseudo R^2^ | 0.002 |
| LR χ^2^ (p-value) | 12.83 (0.999) |
| ***Radius caliper (0.1)*** | |
| Mean absolute bias | 2.2 |
| Pseudo R^2^ | 0.002 |
| LR χ2 (p-value) | 13.41 (0.921) |
| ***Radius caliper (0.001)*** | |
| Mean absolute bias | 1.3 |
| Pseudo R^2^ | 0.001 |
| LR χ2 (p-value) | 6.44 (0.999) |
| **Kernel** | |
| Mean absolute bias | 1.2 |
| Pseudo R^2^ | 0.001 |
| LR χ^2^ (p-value) | 7.37 (1.00) |
| ***Stratification*** | |
| Mean absolute bias | 2.7 |
| Pseudo R2 | 0.013 |
| LR χ2 (p-value) | 82.91 (0.000) |
