# Supplementary material for: The impact of nutritional supplement intake on diet behavior and obesity outcomes
Source: PLoS One. 2017 Oct 9;12(10):e0185258. doi: 10.1371/journal.pone.0185258 (PMC5633155; doi:10.1371/journal.pone.0185258)
Supplement: S3 Table — (DOCX) [file pone.0185258.s003.docx]

**Table:** Reduction in Self-selection Bias and Covariate Balancing

|  |  | **Mean** | |  |  | |  |
| --- | --- | --- | --- | --- | --- | --- | --- |
| **Variable** | **Unmatched/Matched** | **Treated** | **Control** | **% bias** | **% reduction bias** | | **t-test** |
| ***Diet Quality*** | | | | | | | |
| HEI-Total | U |  |  |  |  | |  |
|  | M |  |  |  |  | |  |
| HEI-Total vegetables | U | 3.153 | 3.151 | 3 | 44.9 | | 1.05(0.29) |
|  | M | 3.153 | 3.154 | -1.6 |  |  | -0.56 (0.57) |
| HEI-Greens & beans | U | 2.193 | 2.188 | 2.6 | -6.2 | | 0.91 (0.36) |
|  | M | 2.193 | 2.198 | -2.7 |  |  | -0.93 (0.35) |
| HEI-Total fruits | U | 3.071 | 3.066 | 3.7 | -5.7 | | 1.3 (0.19) |
|  | M | 3.071 | 3.076 | -3.9 |  |  | -1.34 (0.18) |
| HEI-Whole fruits | U | 4.107 | 4.099 | 3.6 | -12 | | 1.27 (0.2) |
|  | M | 4.107 | 4.115 | -4 |  |  | -1.38 (0.17) |
| HEI-Whole grain | U | 1.952 | 1.949 | 3.2 | 69.4 | | 1.14 (0.25) |
|  | M | 1.951 | 1.952 | -1 |  |  | -0.34 (0.74) |
| HEI-Dairy | U | 5.575 | 5.576 | -0.2 | -3101.4 | | -0.07 (0.95) |
|  | M | 5.575 | 5.566 | 6 |  |  | 2.11 (0.04) |
| HEI-Seafood & plant protein | U | 2.997 | 2.989 | 6.6 | 29.8 | | 2.34 (0.02) |
|  | M | 2.997 | 3.003 | -4.6 |  |  | -1.59 (0.11) |
| HEI-Fatty acid ratio | U | 3.965 | 3.964 | 1 | -388 | | 0.37 (0.71) |
|  | M | 3.965 | 3.97 | -5 |  |  | -1.72 (0.09) |
| HEI-Sodium | U | 4.351 | 4.344 | 5.3 | 80.8 | | 1.87 (0.06) |
|  | M | 4.351 | 4.349 | 1 |  |  | 0.35 (0.73) |
| HEI-Refined grains | U | 6.78 | 6.772 | 96.3 | 64.9 | | 2.24 (0.03) |
|  | M | 6.78 | 6.777 | 2.2 |  |  | 0.77 (0.44) |
| HEI-Empty calories | U | 11.49 | 11.481 | 2.6 | 15.1 | | 0.93 (0.35) |
|  | M | 11.49 | 11.498 | -2.2 |  |  | -0.76 (0.45) |
| ***Health Indicators*** | | | | | | | |
| Body mass index | U | 28.685 | 29.299 | -9.2 |  | -3.26 (0.01) | |
|  | M | 28.695 | 28.886 | -2.9 | 69.0 | -1.01 (0.31) | |
| Diabetes | U | 0.134 | 0.115 | 5.5 | 15 | 1.97 (0.05) | |
|  | M | 0.133 | 0.149 | -4.7 |  | -1.54 (0.12) | |
| Blood pressure | U | 0.984 | 0.983 | 0.7 | -90 | 0.25 (0.81) | |
|  | M | 0.984 | 0.982 | 1.3 |  | 0.45 (0.65) | |
| ***Demographics*** | | | | | | | |
| Male | U | 0.4388 | 0.545 | -21.4 | 97.2 | | -7.61 (0.00) |
|  | M | 0.439 | 0.436 | 0.6 |  | | 0.2 (0.84) |
| Age | U | 55.392 | 46.159 | 53.8 | 93.7 | | 19.11 (0.00) |
|  | M | 55.383 | 55.968 | -3.4 |  | | -1.16 (0.25) |
| White | U | 0.558 | 0.401 | 31.9 | 100 | | 11.33 (0.00) |
|  | M | 0.558 | 0.558 | 0 |  | | 0 (1.0) |
| Hispanic | U | 0.099 | 0.119 | -6.7 | 85.7 | | -2.38 (0.02) |
|  | M | 0.099 | 0.098 | 0.3 |  | | 0.1 (0.92) |
| Other race | U | 0.171 | 0.248 | -18.8 | 85.7 | | -6.66 (0.00) |
|  | M | 0.171 | 0.182 | -2.7 |  | | -0.99 (0.32) |

| Citizen | U | 0.925 | 0.828 | 30 | 97.4 | 10.55 (0.00) |
| --- | --- | --- | --- | --- | --- | --- |
|  | M | 0.925 | 0.928 | -0.8 |  | -0.33 (0.74) |

| High school | U | 0.233 | 0.257 | -5.6 | 52.8 | -1.98 (0.05) |
| --- | --- | --- | --- | --- | --- | --- |
|  | M | 0.232 | 0.221 | 2.6 |  | 0.93 (0.3) |
| Graduate | U | 0.254 | 0.133 | 31.2 | 94.8 | 11.16 (0.00) |
|  | M | 0.254 | 0.248 | 1.6 |  | 0.5 (0.62) |
| Married | U | 0.624 | 0.584 | 8.1 | 80 | 2.89 (0.00) |
|  | M | 0.624 | 0.616 | 1.6 |  | 0.57 (0.57) |
| Divorced | U | 0.259 | 0.209 | 11.8 | 73.9 | 4.19 (0.00) |
|  | M | 0.259 | 0.272 | -3.1 |  | -1.02 (0.31) |

| Household size | U | 2.79 | 3.399 | -37.7 | 95 | -13.35 (0.00) |
| --- | --- | --- | --- | --- | --- | --- |
|  | M | 2.791 | 2.822 | -1.9 |  | -0.7 (0.48) |
| HHInc2 | U | 0.217 | 0.229 | -3 | 47.1 | -1.08 (0.00) |
|  | M | 0.217 | 0.224 | -1.6 |  | -0.56 (0.58) |
| HHInc3 | U | 0.194 | 0.191 | 0.7 | 69.4 | 0.25 (0.81) |
|  | M | 0.194 | 0.193 | 0.2 |  | 0.07 (0.94) |
| HHInc4 | U | 0.099 | 0.077 | 7.8 | 84.7 | 2.76 (0.01) |
|  | M | 0.099 | 0.11 | -1.2 |  | -0.38 (0.7) |
| HHInc5 | U | 0.166 | 0.091 | 22.5 | 93.8 | 8.05 (0.00) |
|  | M | 0.165 | 0.161 | 1.4 |  | 0.43 (0.66) |

| ***Lifestyle*** |
| --- |

| Food stamps | U | 0.172 | 0.305 | -28.3 | 90.2 | -9.97 (0.00) |
| --- | --- | --- | --- | --- | --- | --- |
|  | M | 0.172 | 0.185 | -2.8 |  | -1.14 (0.25) |
| Smoker | U | 0.457 | 0.513 | -10.3 | 99.3 | -3.63 (0.00) |
|  | M | 0.457 | 0.456 | 0.1 |  | 0.03 (0.98) |
| Alcohol | U | 0.706 | 0.719 | -2.5 | 9.1 | -0.9 (0.37) |
|  | M | 0.705 | 0.694 | 2.3 |  | 0.8 (0.42) |
| Very active | U | 0.208 | 0.184 | 5.7 | -7.9 | 2.04 (0.04) |
|  | M | 0.204 | 0.178 | 6.2 |  | 2.25 (0.03) |
